# The triad of erectile dysfunction, hypogonadism and the metabolic syndrome

**DOI:** 10.1111/j.1742-1241.2008.01696.x

**Published:** 2008-05-01

**Authors:** R Shabsigh, S Arver, K S Channer, I Eardley, A Fabbri, L Gooren, A Heufelder, H Jones, S Meryn, M Zitzmann

**Affiliations:** 1 Division of Urology, Maimonides Medical Center, Columbia University Brooklyn, NY, USA; 2 Center for Andrology and Sexual Medicine, Karolinska University Hospital/Huddinge Stockholm, Sweden; 3 Royal Hallamshire Hospital Sheffield, UK; 4 Department of Urology, St James University Hospital Leeds, UK; 5 Department of Endocrinology, Universita di Roma Tor Vergata Rome, Italy; 6 Department of Endocrinology, Vrije Universiteit medical center Amsterdam, The Netherlands; 7 Endocrine Clinic & Research Unit Munich, Germany; 8 University of Sheffield Medical School Sheffield, UK; 9 Institute for Medical Education, University of Vienna Vienna, Austria; 10 Institute of Reproductive Medicine Münster, Germany

## Abstract

**Aim:**

To identify the relationship of erectile dysfunction, hypogonadism and the metabolic syndrome in the context of men's health.

**Methods:**

An Expert Panel Meeting was held in December 2006 in Vienna, Austria. In addition a comprehensive literature search was conducted.

**Results:**

Men have a higher incidence of cardiovascular events than women of similar ages which has led to the belief that testosterone is a risk factor for cardiovascular disease in men. The latter hypothesis is no longer tenable. On the contrary, low testosterone levels are associated with (visceral) obesity, the metabolic syndrome, diabetes mellitus, cardiovascular disease and erectile dysfunction (ED). Testosterone therapy does not lead to an increased incidence of cardiovascular disease or events such as myocardial infarction, stroke or angina. Until recently (visceral) obesity, the metabolic syndrome, diabetes mellitus, cardiovascular disease and ED were viewed as more or less independent entities affecting the ageing male. It was not recognised that hypogonadism is a common denominator. With a more integrative approach to the health situation of middle-aged and elderly men, these conditions appear closely interrelated in their manifestations, hypothetically in their aetiology, diagnostic strategy and also their treatment.

**Conclusion:**

Improving sexual health is a portal to identify health hazards and improving men's health. Appropriate diagnosis and medical work up of men presenting with sexual symptoms may have the benefit of the diagnosing and treating other important conditions, such as obesity, diabetes, hypertension and hyperlipidaemia.

**Review Criteria**

The original premise for this review arose from an Expert Panel Meeting held in December 2006 in Vienna, Austria and sponsored by Bayer-Schering AG, Berlin, Germany. The panel discussed whether the low testosterone levels found in men with the metabolic syndrome are an essential element of the syndrome. Further, it was discussed that sexual dysfunction in middle-aged and elderly men should be viewed in the context of their general health.

**Message for the Clinic**

When middle-aged and elderly men seek medical advice for sexual dysfunction, their complaints should no longer be viewed as a single diagnostic/therapeutic entity. Sexual dysfunction is usually an expression of impairment of general health, not rarely the metabolic syndrome. Diagnosis and treatment should include assessment of the components of the metabolic syndrome, which are (visceral) obesity, hypertension, insulin resistance and diabetes mellitus and dyslipidaemia. Thus sexual dysfunction should be a portal to men's health.

There has traditionally been a widely held belief that testosterone is a risk factor for cardiovascular disease in men. This belief was based on the observation that men have both a higher incidence of cardiovascular events and higher testosterone levels than women of similar ages. However, few, if any, recent observations support a causal relation between higher testosterone levels and heart disease (1–3). On the contrary, several studies suggest that higher testosterone levels are associated with a more favourable risk effect on the risk of cardiovascular disease (4,5). Studies of testosterone therapy have not demonstrated an increased incidence of cardiovascular disease or events such as myocardial infarction, stroke or angina (6,7). Evidence that testosterone therapy may be beneficial for men with cardiac disease is becoming stronger (8,9).

This paper will analyse the interrelationship between the metabolic syndrome, diabetes mellitus, cardiovascular disease, (visceral) obesity, erectile dysfunction (ED) and hypogonadism in (ageing) men. Until recently, they were viewed as more or less independent entities befalling the ageing male, if necessary, treated by various medical specialties. With a more integrative approach to the health situation of middle-aged and elderly men, these conditions appear closely interrelated in their manifestations, hypothetically in their aetiology, diagnostic strategy and also their treatment. This paper takes the position that (improving) sexual health is a portal to identify health hazards and improving men's health. Appropriate diagnosis and medical work up of men presenting with sexual symptoms may have the benefit of the diagnosing and treating other important conditions, such as obesity, diabetes, hypertension and hyperlipidaemia.

## The epidemic of obesity and cardiovascular disease and diabetes: the metabolic syndrome

Obesity is a condition that is reaching epidemic proportions in both the developed and the developing world (10). A preferential accumulation of intra-abdominal fat is associated with hypertension, dyslipidaemia [elevated levels of cholesterol, of triglycerides, of low-density lipoproteins (LDLs) and low levels of high-density lipoproteins (HDLs)], impaired glucose tolerance with hyperinsulinaemia, a cluster known as the ‘metabolic syndrome’ (11–13) (Table 1). There is a debate in the literature whether combining these components or conditions has an added diagnostic or prognostic value. Meanwhile, there are three main definitions of the metabolic syndrome. These definitions overlap but differ in the points of emphasis of the components (14–16). The metabolic syndrome is not an end disease by itself but its rather a pathway to disease. A clinical investigation analysing 11 prospective European cohort studies of 6156 men with a median follow-up of 8.6 years found that non-diabetic persons with the metabolic syndrome had increased risk of death of all causes as well as cardiovascular disease (17). The West of Scotland Coronary Prevention Study followed 6000 men for periods over 5 years and found that men with four or five features of the metabolic syndrome had a 3.7-fold increases in coronary heart disease events and, even more strikingly, a 24.5-fold increase in new-onset diabetes (18).

**Table 1:** Definitions of the metabolic syndrome

	At least two of		
		
	WHO*	NCEP†	IDF‡
BMI	≥ 0.30		≥ 30
Waist circumference (cm)	≥ 94	> 102	≥ 94
Waist hip ratio	> 0.90		> 0.90
			At least two of
Triglycerides (mg/dl)	≥ 150	≥ 150	≥ 150
HDL-cholesterol (mg/dl)	< 35	< 40	< 35
Blood pressure (mmHg)	≥ 140/90 or medication	≥ 130/85 or medication	≥ 140/90 or medication
Fasting glucose (mg/dl)	≥ 110	≥ 110	
Fasting insulin	Upper quartile of non-diabetic		Insulin resistance

* World Health Organization, *Diabetes Med* 1999; 16: 442–3.

† National Cholesterol Education Program, *JAMA* 2001; 285: 2486–97.

‡ International Diabetes, *J Urol* 2005; 174: 827–34. BMI, body mass index; HDL, high-density lipoprotein.

## Epidemiological studies linking risk factors of cardiovascular disease to low testosterone levels

Initially cross-sectional studies but later also longitudinal studies were able to confirm that low testosterone levels and sex hormone-binding globulin (SHBG) were predictive of the metabolic syndrome, not only in obese men but also in men with a body mass index (BMI) < 25 kg/m^2^ (19–21). The same applied to the risks of developing diabetes mellitus type 2 (22–24). The association between low testosterone/low SHBG levels and the metabolic syndrome is beyond any reasonable doubt now. The cause and effect relation remains, however, a subject of further study. The following will examine the relationship between testosterone on the one hand and the metabolic syndrome in greater detail. It is becoming clear that the relationship is in fact a two way street: low levels of testosterone predispose to (visceral) adiposity and (visceral) adiposity suppresses the production of testosterone.

## The relationship between hypogonadism and insulin resistance and diabetes mellitus

Cross-sectional epidemiological studies have reported a direct correlation between plasma testosterone and insulin sensitivity, and low testosterone levels are associated with an increased risk of type 2 diabetes mellitus in men (21,24–26), for review: (27,28). A study in untreated hypogonadal Klinefelter patients using the hyperinsulinaemic euglycaemic clamp technique found hyperinsulinaemia and insulin resistance in these patients (29).

A recent study demonstrated a positive correlation between serum testosterone levels and insulin sensitivity in men across the full spectrum of glucose tolerance (30). In this study, men with hypogonadal testosterone levels were twice as insulin resistant as their eugonadal counterparts, and 90% fulfilled criteria for the metabolic syndrome.

## The suppressive effect of adipose tissue on the synthesis of testosterone

The adipocyte functions as an endocrine cell, producing and secreting adipocytokines/adipokines of which leptin is a prominent member. Leptin receptors are present on the Leydig cell and inhibit the testosterone generated by administration of human chorionic gonadotropin (31,32). This finding was supported by studies that found a negative correlation between adiposity, insulin and leptin on the one hand and testosterone levels on the other (33,34).

Hyperinsulinaemia as encountered in insulin resistance might impair testosterone secretion by the Leydig cell, maybe directly as there are insulin receptors on the Leydig cell (35). Conversely, in an elegant study (35). Insulin resistance impairs basal and human chorionic gonadotropin (hCG)-stimulated testosterone secretion from the Leydig cell. Overcoming the hyperglycaemia with the hyperinsulinaemic euglycaemic clamp technique led to a rise of plasma testosterone. This study showed an effect of testosterone on insulin sensitivity within 48 h, so this was not an indirect effect mediated through changes in body composition.

It has also been found in obese men that that there is an attenuated pulse amplitude of luteinizing hormone (LH) while the LH pulse frequency is unaffected, thus producing a less strong stimulation of testicular testosterone production (36,37).

## Metabolic effects of (rather) acute androgen deprivation in men with prostate cancer

Prostate cancer is an androgen-dependent malignancy and, consequently, androgen deprivation treatment (ADT) has a role to play in its management (38). Several studies have documented the deterioration of metabolic control upon ADT (39–49) in diabetic and non-diabetic men. Collectively, these studies have found that ADT leads to an increase in fat mass, an increase in plasma insulin, decreased insulin sensitivity (48), increased levels of glycosylated haemoglobin, an increase in plasma cholesterol, LDL-cholesterol and triglycerides but also an increase in plasma HDL-cholesterol, which contrasts with the low HDL-cholesterol levels found in the metabolic syndrome.

A recent study analysed the effects of GnRH agonist administration to men with prostate cancer; 73,196 Medicare patients ≥ 66 years who had locoregional prostate cancer between 1992 and 1999 were followed through 2001. The study assessed whether treatment with GnRH agonists or was associated with diabetes, coronary heart disease, myocardial infarction and sudden cardiac death. GnRH agonist therapy was associated with increased risk of incident diabetes [adjusted hazard ratio (HR), 1.44; p < 0.001], coronary heart disease (adjusted HR, 1.16; p < 0.001), myocardial infarction (adjusted HR, 1.11; p = 0.03) and sudden cardiac death (adjusted HR, 1.16; p = 0.004). Therefore, GnRH agonist treatment should be weighed against these potential risks (44).

At the cellular levels there is now insight into the effects of androgen deprivation/administration on fat mass. Testosterone regulates lineage determination in mesenchymal pluripotent cells by promoting their commitment to the myogenic lineage and inhibiting their differentiation into the adipogenic lineage through an androgen receptor-mediated pathway. The observation that differentiation of pluripotent cells is androgen dependent provides a unifying explanation for the reciprocal effects of androgens on muscle and fat mass in men (50,51).

Prostate cancer patients treated with ADT need to be monitored with reference to possible metabolic consequences of ADT. The survival of patients with prostate cancer has improved considerably. Cause of death in prostate cancer patients is no longer mainly because of the malignancy but to other conditions (52). Therefore, it has become increasingly important to address side effects of treatment timely and appropriately (53).

## Testosterone in men with diabetes mellitus type 2

Similar to studies in men with the metabolic syndrome, there is an inverse relationship between testosterone levels and diabetes. This relationship has been established and confirmed since the mid-1980s. A recent study reported not only low levels of testosterone but also symptoms thereof in men with diabetes stressing the clinical relevance of the association (54). Men with diabetes have lower testosterone levels compared with men without a history of diabetes, even without adiposity (24).

A recent systematic review and meta-analysis of cross-sectional studies indicated that testosterone level was significantly lower in men with type 2 diabetes [mean difference, −76.6 ng/dl; 95% confidence interval (CI), −99.4 to −53.6]. Similarly, prospective studies showed that men with higher testosterone levels (range: 449.6–605.2 ng/dl) had a 42% lower risk of type 2 diabetes (relative risk, 0.58; 95% CI: 0.39–0.87). In addition, several large prospective studies have shown that low testosterone levels predict development of type 2 diabetes in men (22,23,55).

A recent study analysing the effects of testosterone administration to 24 hypogondal men (10 treated with insulin) over the age of 30 years with type 2 diabetes found that testosterone replacement therapy reduced insulin resistance (as measured by homeostatic model index) and improved glycaemic control in hypogonadal men with type 2 diabetes (56). There are indications now that the beneficial effects of testosterone on the metabolic syndrome are related to their impact on body composition and this effect will only occur in the longer-term (57,58) 6–12 months of testosterone administration. This might explain why shorter term studies (3 months) do not show many favourable effects (56).

## New insights into the relationship of androgens and erectile dysfunction

Erectile response in mammals is centrally and peripherally regulated by androgens. Severe hypogonadism in men usually results in loss of libido and potency which can be restored by androgen administration. The original insights into the mechanisms of action of androgens on sexual function indicated a prominent role of testosterone on sexual interest while the effects of testosterone on erectile function were less apparent from these investigations (59). There is growing insight that testosterone has profound effects on tissues of the penis involved in the mechanism of erection and that testosterone deficiency impairs the anatomical and physiological/biochemical substrate of erectile capacity, reversible upon androgen treatment. An improvement of nocturnal erections [tumescence and rigidity, spontaneous and sexually related erections was found in men with androgen deficiency upon treatment with transdermal testosterone (60) and eugonadal circumstances].

Several studies have indicated that the administration of phosphodiesterase inhibitor type-5 (PDE-5) inhibitors is not always sufficient to restore erectile potency in men, and that administration of testosterone improves the therapeutical response to PDE-5 inhibitors considerably (61,62). There is increasing insight not to view ED as a single entity but as part of the ageing process. Circulating levels of testosterone are closely related to manifestations of other aetiological factors in ED, such as atherosclerotic disease and diabetes mellitus. The latter are correlated with lower-than-normal testosterone levels.

Erectile difficulties provide often a window into the presence of pathology in these areas. Rather than a disease in itself, ED is, particularly in elderly men who have enjoyed normal sexual function earlier in life, a manifestation of pathologies of the biological systems involved in erectile function (63).

Animal experiments and, to a much more limited degree, human observations suggest that androgens are necessary to maintain the integrity of the anatomical structures of the penile erectile tissue and, further, that androgens are significant in the biochemical mechanisms subserving penile erection.

There is now ample evidence from animal studies that androgen deprivation produces changes in the histological properties of penile structures. Shabsigh (64) could demonstrate that castration caused apoptosis in the rat corpus cavernosum smooth muscle after only 3 days. Administration of testosterone restored DNA synthesis already after 4 days. In a rat model Shen et al. [reviewed in (65)] demonstrated that androgen deprivation leads to loss of elastic fibres in the tunica albuginea and of smooth muscle fibres in the corpus cavernosum [reviewed in (65)] which were replaced by collagenous fibres in both structures. Singh et al. [reviewed in (65)] found that the mesenchymal pluripotent cells follow a myogenic lineage or adipogenic lineage depending on circulating levels of testosterone, confirmed by Traish et al. [reviewed in (65)].

Traish and coworkers [reviewed in (65)] demonstrated that even a 50% reduction in circulating testosterone reduced intracavernosal blood pressure which was not enhanced by administration of the PDE-5 inhibitor vardenafil. Zhang et al. (66) showed that also continuous administration of tadalafil could not enhance erectile response in castrated animals. Nitric oxide synthase and arginase activities in the corpus cavernosum were not significantly affected by the reduction in circulating testosterone confirmed in human tissue by Morelli et al. (67). Yassin and Saad (68) showed that adequate testosterone treatment can restore venous leakage in the corpus cavernosum which is a frequent etiological factor in ED in elderly men.

Schiavi and Rehman (69), based on their clinical experience, hypothesised that the threshold for the biological actions of testosterone might be higher in elderly men compared with young men. Their hypothesis was recently convincingly experimentally confirmed by Gray et al. (70) showing that, compared with younger men, elderly men require higher levels of circulating testosterone for libido and erectile function.

## Erectile dysfunction is a ‘portal’ to men's health

The above has demonstrated a close relationship between ailments frequently occurring in the ageing male (visceral obesity, cardiovascular disease, diabetes mellitus and ED) on the one hand and hypogonadism on the other. In view of this close relationship, late onset hypogonadism is probably an expression of poor health with a high-risk profile for debilitating diseases, as may be concluded from a recent study. Low testosterone levels were associated with increased mortality in male veterans (71). This association between sex steroids and all-cause and cause-specific mortality could not be confirmed in Massachusetts Male Aging Study (72).

Men usually are in denial of ailments at all ages, but certainly also when they are ageing. Erectile function is viewed by almost all men as a significant component of quality of life (73) and erectile difficulties (ED) may be a reason to seek medical advice. Several studies document now that there is a high concordance between the causes of ED and the causes of cardiovascular disease, this indirectly by demonstrating that there is an elevated prevalence of the metabolic syndrome and insulin resistance in a population of men with ED when compared with a general population of men (74). The authors argue that the ultimate goal therefore must be not only to treat the erectile problem but also to diagnose and adequately (aggressively) treat any cardiac risk factors that may be found.

The prestigious Massachusetts Male Aging Study equally revealed that ED was predictive of the metabolic syndrome, although only in men with BMI < 25. This finding also supports the notion that waist circumference or waist/hip ratio are more reliable indicators of the metabolic syndrome that BMI. This study supports the idea that ED may provide a warning sign and, at the same time, an opportunity for early intervention in men otherwise considered at lower risk for the metabolic syndrome and subsequent cardiovascular disease (75). The Massachusetts Male Aging Study (MMAS) has estimated the frequency of ED progression and remission among ageing men, and assessed the relation of progression/remission to demographics, socioeconomic factors, comorbidities and modifiable lifestyle characteristics. Natural remission and progression occur in a substantial number of men with ED. Age and BMI were associated with progression and remission of ED. Interventions were non-pharmacological which apparently impacted on remission and delaying progression of ED. The association of BMI with remission and progression, and the association of smoking and health status with progression, offer potential avenues for facilitating remission and delaying progression using non-pharmacological intervention. The benefits of such interventions for overall men's health may be far reaching and support the view that sexual health is a portal to men's health (76). Lifestyle changes are associated with improvement in sexual function in about one-third of obese men with ED at baseline. Weight loss and increased physical activity, and lower caloric intake, with a Mediterranean type of diet, appeared to have a favourable effect on erectile and endothelial functions in obese men (77,78). It is recommendable that these lifestyle changes are continued lifelong.

Shabsigh et al. (79) have eloquently argued that ED can calculate men's health risks (Table 2). The men's health calculator is intended to raise awareness about cardiovascular disease and diabetes risk. It is intended to be easy, short, quick and suitable for both print and internet. The four questions address different aspects of body functions and health. These questions were derived from the Men's Attitudes to Life Events and Sexuality (MALES) study that included a large number of subjects in eight countries. The questions function with their linguistic face meaning without further explanation. The first question is self-rating of perceived state of health. The second question is inquiring about having severe ED. This could be physiologically hypothesised as surrogate for vascular health. The third question is about having a good relationship with the partner. This could be hypothesised to be surrogate for mental and behavioural health. The fourth question is about waist circumference, which can be hypothesised to be a surrogate for metabolic health. Elements in the calculation of health risks (hypertension, diabetes, angina or hyperlipidaemia) in men presenting with ED are: health status on a scale of 1–7 (1 = excellent, 7 = poor), waist circumference, severity of ED, presence/absence of a sexual partner. The calculation produces scores of ranges of 1–7. If the score is 1.5–2.5 = medium risk (30–59% probability); ≥ 2.5 = high risk (≥ 60% probability of having the condition) and < 1.5 = low risk (< 30% probability).

**Table 2:** Erectile dysfunction (ED) can calculate men's health risks: the MALES study

Variable	Answer	Values	Score
Health status		0.5	
Severe ED		1	
Sexual partner		−1.5	
Waist size: 86–91 cm		1	
Waist size: 96–101 cm		2	
Waist size over 106 cm		2	
		Total	

Y = f (health status, waist size, ED severity and sexual partner) where Y = 0, 1 (no disease/disease). 90% sample = 349. 10% holdout sample = 40.

Risk for hypertension, diabetes, angina or hyperlipidaemia. Score ranges from 0 to 7.

≥ 2.5 = high risk (≥ 60% probability of having the condition).

1.5–2.5 = medium risk (30–59% probability).

< 1.5 = low risk (< 30% probability).

Score of our patient: 3 = high risk.

If he had not had ED: 2 = medium risk.

## Conclusions

Sexual health may be the portal to men's health. Lifestyle changes involving more exercise and a lower caloric intake, with a Mediterranean type of diet, appear to improve sexual function (77,78). It is recommendable that these measures are lifelong.

Hypogonadism and ED are epidemiologically associated with and may predict metabolic syndrome and diabetes type 2. However, there are significant limitations and knowledge gaps. Most evidence of the link of hypogonadism and metabolic syndrome is observational. Interventional studies are needed to determine the relationship between testosterone and diabetes mellitus and the metabolic syndrome to assess the benefit/risk ratio of testosterone replacement therapy in men with the hypogonadism, diabetes and the metabolic syndrome. Treatment of ED may entail testosterone administration (80) and these interventional studies may provide an opportunity to determine therapeutic and preventive feasibility, benefits and justification of testosterone administration on the closely interrelated ailments of ED and the metabolic syndrome of which epidemiologically hypogonadism is a correlate.
